# Characterization of the sex-specific pattern of angiogenesis and lymphangiogenesis in aortic stenosis

**DOI:** 10.3389/fcvm.2022.971802

**Published:** 2022-09-12

**Authors:** Lara Matilla, Ernesto Martín-Núñez, Mattie Garaikoetxea, Adela Navarro, Julieta Anabela Vico, Vanessa Arrieta, Amaia García-Peña, Amaya Fernández-Celis, Alicia Gainza, Virginia Álvarez, Rafael Sádaba, Natalia López-Andrés, Eva Jover

**Affiliations:** Cardiovascular Translational Research, Navarrabiomed, Hospital Universitario de Navarra (HUN), Universidad Pública de Navarra (UPNA), IdiSNA, Pamplona, Spain

**Keywords:** aortic stenosis, sex, valve interstitial cells, angiogenesis, lymphangiogenesis

## Abstract

**Objective:**

We aim to analyze sex-related differences in angiogenesis and lymphangiogenesis in aortic valves (AVs) and valve interstitial cells (VICs) from aortic stenosis (AS) patients.

**Approach and Results:**

Totally 230 patients (59% men) with severe AS undergoing surgical valve replacement were recruited. The density of total neovessels was higher in AVs from men as compared to women. Both small and medium neovessels were more abundant in men's AVs. Accordingly, male AVs exhibited higher CD31 and VE-cadherin expressions. The levels of the pro-angiogenic markers, such as vascular endothelial growth factor (VEGF)-A, VEGF receptor (VEGFR)1, VEGFR2, insulin-like growth factor-binding protein-2 (IGFBP-2), interleukin (IL)-8, chemerin, and fibroblast growth factor (FGF)-7, were increased in AVs from men. Transforming growth factor-β expression was higher in male AVs. The expression of antiangiogenic molecules thrombospondin (Tsp)-1, endostatin, and CD36 was upregulated in male AVs, although the levels of Tsp-2, IL-4, IL-12p70, and chondromodulin-1 were similar between both sexes. The number of lymphatic vessels and the expression of the lymphangiogenic markers Lyve-1 and D2-40 was higher in men's AV as well as VEGF-C, VEGF-D, and VEGFR3. Multivariate analyses adjusted for confounders further validated the sex-dependent expression of these targets. VICs isolated from men's AVs secreted higher amounts of the pro-angiogenic factors, VEGF-A, VEGFR1, IGFBP-2, and FGF-7, as well as the pro-lymphangiogenic factors, VEGF-C, VEGF-D, and VEGFR3, than women without changes in antiangiogenic markers.

**Conclusion:**

Our data show that aberrant angiogenic and lymphangiogenic cues are over-represented in male AVs. Importantly, the VIC is a relevant source of multiple morphogens involved in angiogenesis and lymphangiogenesis likely endowing the AV of men with the predominant calcific AS phenotypes.

## Highlights

- Angiogenesis and lymphangiogenesis processes are different in men and women with AS, being over-represented in men.
- AVs from men presented a higher density of blood and lymphatic vessels accompanied by higher expression of pro-angiogenic and lymphatic factors, while in women angiogenesis might result from the downregulation of physiologic angiogenic inhibitors.
- VICs are a relevant source of pro-angiogenic and lymphangiogenic molecules, especially in men.

## Introduction

Aortic stenosis (AS) is a major health problem that affects 2 to 7% of individuals older than 65 years (1). There are currently no pharmacological strategies to prevent, delay, or reverse AS (2). Inflammation, fibrosis, apoptosis, calcification, and angiogenesis are fundamental to the progression of AS and endow the aortic valve (AV) with fibro-calcific phenotypes (3). AS is more prevalent in men, and recent evidence shows clear sex-specific differences in clinical presentation and patient management (4, 5). We have recently described that for the same AS severity, AVs and valve interstitial cells (VICs) from men presented more inflammation, apoptosis, calcification, and diminished extracellular matrix remodeling than AVs and VICs from women (6).

The pathophysiological role of angiogenesis and lymphangiogenesis in AS remains unknown. Valve avascularity is seemly abrogated in AS (7) and the extent of neovascularization is well-correlated with the burden of the disease (8, 9). Angiogenic factors have been found to co-localize with osteopontin and osteocalcin in the late stage of heavily thickened and calcified atherosclerotic plaques (8, 10). It seems now to become evident that angiogenesis may promote calcific AS (11). Neovascularization is speculated to be a maladaptive response to the extensive valve cusp thickening during fibrosclerotic stages and the enhanced requirements of oxygen supply (12). It might also contribute to perpetuating osteogenesis in advanced calcified AVs similarly to that occurring during endochondral bone formation (13, 14). Contradictory data reveal an association between neovessel formation and inflammation (12, 15). Pro-angiogenic vascular endothelial growth factor (VEGF)-A and its receptors VEGFR1 and VEGFR2 have been associated with inflammation in AS patients (12). However, some recent data suggest that aberrant angiogenesis may instead precede inflammation (15). Furthermore, lymphatic vessels have been detected in AVs from AS patients (16–18). Lymphangiogenic growth factors, such as VEGF-C, VEGF-D, and VEGFR3, are over-expressed in AVs from AS patients (18).

Owing to pericyte-like differentiation, the VIC may support neovascularization in AS (19, 20) but also be a major contributor to the myofibroblast (21) and osteoblast pools (22–24). Interestingly, VICs secrete VEGF-A (3, 20, 25) and are one of the proposed sources of pro-lymphangiogenic factors VEGF-C and VEGF-D and its receptor VEGFR3 (18). Noteworthy, early transcriptomic analyses revealed over-represented angiogenic pathways in male porcine VICs (26). A recent publication has also shown that the angiogenic secretome varies with sex in porcine VICs (27). However, the information about sex-specific differences in angiogenesis and lymphangiogenesis in human AS is scarce and remains understudied. Our work aimed to provide a thorough descriptive characterization of sex-related angiogenic and lymphangiogenic signatures in patients with AS. To that, histological, molecular, and cellular approaches have been employed.

## Materials and methods

### Clinical cohort

This study, prospective and observational, involved a total of 230 patients with severe AS (28) referred to Hospital Universitario de Navarra for surgical AV replacement from June 2013 to October 2020. AS was defined as AV area ≤ 1 cm^2^ and/or transaortic mean pressure gradient > 40 mm Hg. The presence of moderate or severe concomitant valvular disease, malignant tumor, infective endocarditis, diabetes mellitus, and chronic inflammatory diseases were exclusion criteria for the study.

All patients were evaluated by transthoracic echocardiography. Venous blood was drawn on admission for surgery for Measurement of brain natriuretic peptide (BNP) and other routine laboratory parameters.

Informed consent was obtained from each patient. The study protocol conforms to the ethical guidelines of the 1975 Declaration of Helsinki as reflected in a previous approval by the institution's human research committee (Comité Ético de Experimentación Clínica. Gobierno de Navarra, Departamento de Salud; Ethics numbers 17/2013 and PI2019/59).

AV leaflets harvested from AS patients were dissected into three pieces. One “healthier” part was dedicated to VICs isolation (*n* = 26) to guarantee the *in vitro* viability and expansion of the VICs with physiologic-like phenotypes; another one was paraffin-embedded and the last one was used for protein and RNA extractions. The dissection of the samples dedicated to molecular and histological analyses (*n* = 230) was aimed to get equal parts of the leaflet/s with comparable amounts of calcific, fibrotic, and “healthy” tissue to avoid biasing ulterior analyses. Different remodeling or pathological processes have been described depending on the location of the leaflet (e.g., different mechanobiological stimulation) during the progression of the AS. Accordingly, no independent leaflets were used for different purposes. Moreover, all AVs were processed and analyzed without knowing the sex of the donor to avoid biased results.

### Cell isolation and culture

Human VICs were isolated from 26 AVs (14 men and 12 women from the whole cohort of 230 AVs processed in this manuscript) obtained during surgical AV replacement. VICs from each patient were isolated and individually assayed, as previously described (29). In brief, AVs were minced and enzymatically digested with buffered-collagenase type 2 (240 U/mg) for 1 h and were pelleted by centrifugation. VICs were cultured in DMEM F-12 medium (Gibco) supplemented with 20% fetal bovine serum (FBS) (Gibco), 1% Penicillin/Streptomycin (Lonza), 5 μg/ml insulin (Sigma Aldrich), and 10 ng/ml of fibroblast growth factor (FGF-2) (Novus Biological) at 37°C and 5% CO_2_ in a saturation humidified incubator (Panasonic). This growing media has been adapted from previous publications (30) and allows for the expansion of VICs upon isolation and until reaching the required cell number for further *in vitro* experiments. The VIC phenotype of isolated cells was confirmed by vimentin and alpha-smooth muscle actin (α-SMA) immunocytochemistry. Experiments were performed in serum-starvation conditions (1% FBS) in multiwell plates (Sarstedt) for 2 days. The aim of serum starvation in our experimental settings is three-fold: first, to induce quiescent-like phenotypes of isolated VICs (30); second, to deplete the content of growth factors (such as VEGFs) (31) in FBS that may mask these released by VICs; and third, to deplete the content of potential physiologic inhibitors of ectopic calcification that has been strongly linked to angiogenesis *in vivo* (32).

### Histology and immunohistochemistry evaluation

Histological determinations in AVs were performed in 5 μm-thick paraffin-embedded serial sections following the protocol of Leica BOND-Polymer Re-fine Detection automatic immunostainer (Leica). All solutions were filled into the bottle-Bond Open Container (Leica) and registered on a computer using the Leica Biosystem program. The immunostaining program protocol includes fixative solution, bond wash solution, blocking with a common immunohistochemistry blocker, and incubation with the following primary antibodies: CD31 (Santa Cruz Biotechnology), VE-cadherin (Abcam), glycophorin-A (Santa Cruz Biotechnology), CD34 (Santa Cruz Biotechnology), D2-40 (Leica), erythroblast transformation-specific related gene (ERG, Roche), VEGF-A (Santa Cruz Biotechnology), VEGFR3 (Santa Cruz Biotechnology), IGFBP-2, Tsp-1, TGF-β, and Lyve-1 (Santa Cruz Biotechnology). After primary antibody incubation, slides were incubated with secondary poly-HRP-IgG. The signal was revealed by using a DAB substrate. Incubation with no primary antibodies was carried out as negative controls.

AV structure, vessels, and inflammatory infiltrates were visualized by hematoxylin/eosin staining (Panreac/Bio-optica). Total vessels, blood vessels, and lymphatic vessels were counted in 168 AS valves and normalized to the surface (mm^2^). For total neovessels, CD31 and VE-cadherin and complementary hematoxylin/eosin staining were used. The presence of blood vessels was assessed in ERG immunostaining and further confirmed by glycophorin-A (detecting erythrocytes) and CD34. Cross-sectional blood vessels were classified according to their size: small (S, with 2 to 3 endothelial cells), medium (M, more than 3 endothelial cells), or hypertrophied (H, with more than 2 to 3 layers of vascular smooth muscle cells), and were normalized to the total number of vessels. Double Lyve-1 with CD31 and CD34 staining and D2–40 staining was performed to identify and count lymphatic vessels. Lyve-1 positive macrophages were excluded from the lymphatic vessel count as reported by Syvaranta et al. (18). All the counting was performed by two different blinded observers using the different immunohistochemistry profiles to reinforce the results.

Histological and immunohistochemistry preparations were imaged using a bright field in an automated image analysis system (Nikon). In brief, arbitrary fields per section were imaged at 50, 100, 200, or 400X magnification, as appropriate. The whole slide was imaged without knowing the sex of the donor and kept for ulterior batch analyses of the amount and type of vessels. Accordingly, no arbitrary fields were imaged and any digital image quantification has been normalized either to the total number of vessels or to the total area of the histological preparation (mm^2^). The most representative images were displayed in the figures along the manuscript.

### ELISA

CD31, VE-cadherin, VEGF-A, VEGFR1, VEGFR2, IGFBP2, IL-8, Chemerin, FGF-7, TGF-β, Tsp-1, Tsp-2, IL-4, IL-12p70, endostatin, VEGF-C, VEGF-D, and VEGFR3 were measured in AVs extracts and cells supernatants according to the manufacturer's instructions (R&D Systems). Known yields of total protein were assayed by ELISA. For the study of secretomes, equal volumes of cell supernatants were loaded upon confirming no differences in cell densities (by protein quantification of the corresponding cell monolayers) among biological replicates.

### Real-time reverse transcription PCR

Total RNA from cells and AVs was extracted with Trizol Reagent (Canvas). The first-strand of cDNA was synthesized according to the manufacturer's instructions (Bio-Rad). Quantitative PCR analysis was performed with SYBR green PCR technology (Bio-Rad) (Chm-1: Forward-GGAGGAGATGCTCTGTTTGG and Reverse-GGAAATAGACGCTGGGAACA; Lyve-1: Forward-GGTTCCAGTGAGCCGACAGT and Reverse-TGCACGAGTTAGTCCAAGTATCAGA; D2-40: Forward-ACCAGTCACTCCACGGAGAAA and Reverse-GGTCACTGTTGACAAACCATCT). Relative quantification was achieved with MyiQ (Bio-Rad) software according to the manufacturer's instructions. Data were normalized to 18S (Forward: CGCCGCTAGAGGTGAAATTC and Reverse: TCTTGGCAAATGCTTTCGC), HPRT (Forward: TTGCTTTCCTTGGTCAGGCA and Reverse: ATCCAACACTTCGTGGGGTC), β-actin (Forward: GCCGCCAGCTCACCAT and Reverse: TCGATGGGGTACTTCAGGGT), and GADPH (Forward: ACCAGCCCCAGCAAGAGCACAAG and Reverse: TTCAAGGGGTCTACATGGCAACTG) levels, and expressed as fold-change relative to men. All PCRs were performed at least in triplicate for each experimental condition.

### Western blot analysis

Aliquots of 10 to 20 μg of total proteins were prepared and electrophoresed from AV extracts on SDS polyacrylamide gels (4–15% polyacrylamide, Mini-PROTEANTGX Stain-Free, BioRad) and transferred to Hybond-C Extra nitrocellulose membranes (BioRad). Membranes were incubated with primary antibodies for CD36 (Santa Cruz Biotechnology) and β-actin (Sigma-Aldrich); and with secondary antibodies for mice and rabbits (GE Healthcare). Blot densitometry analyses were performed using Image Lab software. β-actin and stain free were used as loading controls for normalization and the net band densitometry was expressed as fold changes of arbitrary units (AU). Positive blots were detected with a chemiluminescence method (ECL, Amersham Biosciences) and images acquired with the Chemidoc MP Imaging system (Bio-Rad). All western blots were performed at least in triplicate for each experimental condition. Semiquantitative analyses were performed by band densitometry using the Image Lab software (Bio-Rad).

### Statistical analyses

Patients' data were summarized using frequencies and percentages, means, and standard deviations (SD). Data normality were assessed through Shapiro–Wilk's test and the Kolmogorov–Smirnov test (with Lilliefors *p*-value). Quantitative variables were analyzed by student's *t*–test or the Mann–Whitney U test if the normality was not met. Univariate linear regressions models were fitted for all continuous variables. Categorical variables were expressed as percentages and compared using the χ2 test or the Fisher exact test, as appropriate. The effect of sex on demographic, clinical, and analytic-related variables was assessed in two steps. First, univariate linear regressions models were fitted for all continuous variables. Similarly, univariate logistic regression models were used to estimate the odds ratios of categorical variables. These steps allowed us to identify the variables that differed significantly between sexes and that therefore could be potential confounders. To adjust for the potential confounders found by the univariate analyses, a second analysis step was added where the adjusted effect of sex over the analyzed variables was modeled using linear and logistic multivariate regression models, as appropriate. In brief, based on the magnitude of the effect and the *p*-values calculated in the univariate models, age, statin use, and total cholesterol levels were used as covariates of the multivariate models. For each variable, an adjusted model was fitted to calculate the odds ratio (OR). The absence of multicollinearity was guaranteed by making use of the Variance Inflation Factor for each independent variable. The adjusted *p*-value was therefore calculated as the *p*-value of the sex covariate in the multivariate model that was adjusted for the variables chosen in step one. A *p*-value of < 0.05 was considered statistically significant. All analyses in the clinical cohort were performed using the R statistical package, v. 3.6 (R Foundation for Statistical Computing. Vienna, Austria). GraphPad Software Inc. was used for *in vitro* analyses.

## Results

### Clinical parameters in AS patients

A total amount of 230 patients (59% men) were recruited. Men were significantly younger than women and exhibited higher height and weight, with no differences in body mass index, and lower total cholesterol, as expected according to previous publications (6, 33). Clinical and demographical data of the AS patients included in this manuscript have been summarized in Table 1.

**Table 1:** Clinical and demographical data of AS patients.

**Variable**	**Male**	**Female**	**Total**	***p*–value**
*N (%)*	136 (59)	94 (41)	230	
Age, Mean ± SD	69.7 ± 9.3	73.1 ± 8.4	71.1 ± 9.1	0.003
BMI, Mean ± SD	29.0 ± 4.1	29.2 ± 5.4	29.1 ± 4.7	0.777
DM, *n (%)*	47 (28.8)	28 (25.2)	75 (27.4)	0.581
Renal insufficiency, *n (%)*	9 (5.5)	9 (8.1)	18 (6.6)	0.460
HTA, *n (%)*	115 (70.6)	71 (64.0)	186 (67.9)	0.292
**Drug medicines**				
ACEI, *n (%)*	39 (24.1)	28 (25.5)	67 (24.6)	0.886
ARB, *n (%)*	45 (27.8)	22 (20.0)	67 (24.6)	0.154
Diuretics, *n (%)*	82 (50.3)	63 (56.8)	145 (52.9)	0.325
β-blockers, *n (%)*	53 (32.5)	29 (26.1)	82 (29.9)	0.284
Statins, *n (%)*	120 (73.6)	54 (48.6)	174 (63.5)	<0.001
**Biochemical analyses**				
TGAs (mg/dL), Mean ± SD	109.4 ± 63.3	106.0 ± 39.9	108.1 ± 55.1	0.627
Total cholesterol (mg/dL), Mean ± SD	166.8 ± 37.4	190.7 ± 39.7	176.4 ± 40.0	<0.001
**Echocardiographic parameters**				
Max gradient, Mean ± SD	77.1 ± 19.0	80.9 ± 21.0	78.6 ± 19.9	0.132
Mean gradient, Mean ± SD	49.0 ± 13.2	52.0 ± 14.0	50.2 ± 13.5	0.080
EF %, Mean ± SD	64.3 ± 12.9	66.5 ± 11.4	65.2 ± 12.3	0.161

SD, standard deviation; BMI, body mass index; DM, diabetes mellitus; HTA, artery hypertension; ACEi, Angiotensin-converting enzyme inhibitor; ARB, Angiotensin II receptor blocker; TGA, triglyceride acids; EF, ejection fraction.

### Men-derived AVs present more neovessels than women's

**Figure 1:**
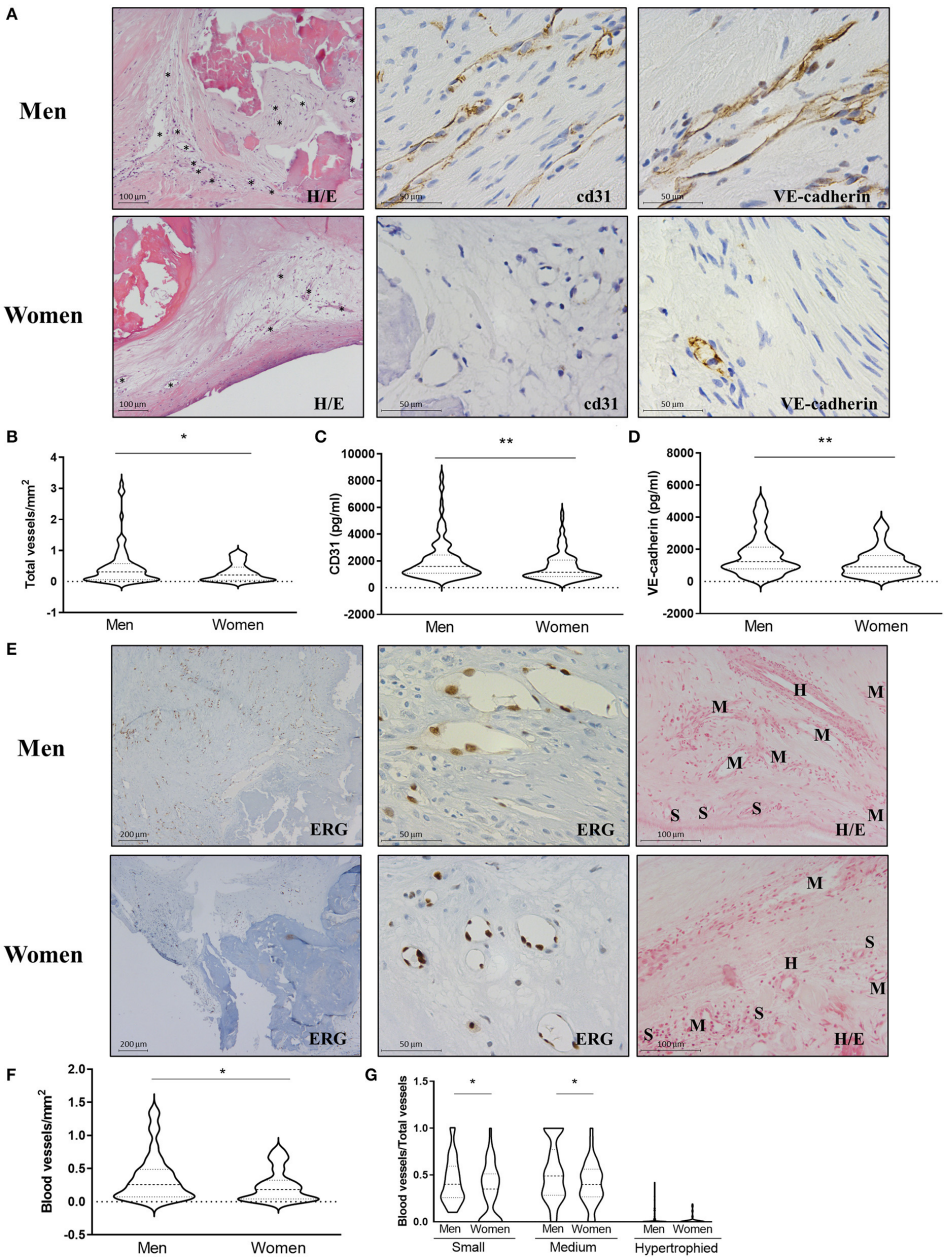
Sex differences in neovessels density in AVs from AS patients. Representative microphotographs of AV sections from men and women AS patients stained for hematoxylin/eosin and immunostained for CD31 and VE-cadherin AV tissue **(A)**. Asterisk indicates neovessels. Total vessel density in AVs from men and women **(B)**. Protein expressions of CD31 and VE-cadherin in tissue homogenates from AVs of AS patients were measured by ELISA **(C,D)**. Representative microphotographs immunostained for the blood vessel marker ERG at low and high magnification and stained for hematoxylin/eosin for blood vessel size **(E)**. Blood vessel density **(F)** according to their size **(G)**. CD31, cluster differentiation 31; VE-cadherin, vascular endothelial cadherin; ERG: ETS-related gene. Different sample sizes were assayed depending on the analytical methods used. *N* = 99 for men and *N* = 69 for women by histology. *N* = 131 for men and *N* = 87 for women by ELISA. ^*^p < 0.05 vs men, ^**^p < 0.01 vs. men.

Neovascularization was found in 73% of the AVs. In men, the frequency was 72%, whereas in women, it was 74%. Neovessel counting in hematoxylin-eosin preparations revealed that AVs from men exhibited more density of neovessels than women's [0.50 ± 0.66 for men vs. 0.29 ± 0.28 for women (vessels/mm^2^)] (Figures 1A,B). Neovascularization was further validated using complementary immunohistochemical and molecular analyses. AVs from men were more positive for CD31 and VE-cadherin than women's (Figure 1A). Accordingly, men AVs exhibited higher CD31 [2,117 ± 1,598 for men vs. 1,579 ± 1,083 for women (pg/ml)] (Figure 1C) and VE-cadherin levels [1,676 ± 1,284 for men vs. 1,177 ± 884 for women (pg/ml)] (Figure 1D) than AVs from women measured by ELISA. Importantly, multivariate analyses adjusting for confounding factors (age, statins treatment, and total cholesterol) further confirmed a lower density of neovessels/mm^2^ (OR = −0.2, *p* = 0.037), CD31 (OR = −532.87, *p* = 0.02), and VE-cadherin expression (OR = −415.31, *p* = 0.0025) in women AVs compared to men's (Table 2).

**Table 2:** Multivariate analyses after adjusting for confounder factors in the study cohort.

**Variable**	**OR^a^ (95% CI)**	**Adjusted**
		***p*–value**
Neovessels/mm^2^	−0.2 (−0.39; −0.01)	0.037
CD31 (pg/mL)	−532.87 (−980.24; −85.49)	0.02
VE–cadherin (pg/mL)	−415.31 (−778.03; −52.59)	0.025
**Angiogenic targets**		
Blood vessels/mm^2^	−0.17 (−0.3; −0.04)	0.01
Small vessels/total vessels	−0.1 (−0.22; 0.03)	0.122
Medium vessels/total vessels	−0.14 (−0.27; 0)	0.05
Hypertrophied vessels/total vessel	−0.02 (−0.05; 0)	0.103
VEGF–A (pg/mL)	−71.5 (−152.83; 9.84)	0.085
VEGFR1 (pg/mL)	−131.76 (−288.47; 24.95)	0.099
VEGFR2 (pg/mL)	−11.54 (−24.91; 1.84)	0.091
IL−8 (pg/mL)	−179.48 (−281.49; −77.46)	0.001
IGFBP−2 (pg/mL)	−77.45 (−177.93; 23.03)	0.131
FGF−7 (pg/mL)	−64.95 (−102.43; −27.47)	0.001
TGF–β (pg/mL)	−57.14 (−85.89; −28.39)	<0.001
Tsp−1 (pg/mL)	−3.85 (−5.93; −1.77)	<0.001
Tsp−2 (pg/mL)	−368.12 (−789.43; 53.19)	0.087
IL−4 (pg/mL)	−1.37 (−7.5; 4.76)	0.661
IL−12p70 (pg/mL)	−19.24 (−34.58; −3.9)	0.014
Endostatin (pg/mL)	−197.81 (−356.02; −39.6)	0.014
CD36 (A.U.)	−0.41 (−0.73; −0.08)	0.014
Chm−1 (mRNA)	−0.01 (−0.04; 0.03)	0.746
**Lymphangiogenic targets**		
Lymphatic vessels/mm^2^	−0.02 (−0.04; 0)	0.045
Lyve−1 (mRNA)	−0.92 (−3.32; 1.48)	0.453
D2–40 (mRNA)	−0.01 (−0.02; 0)	0.041
VEGF–C (pg/mL)	−79.05 (−138.38; −19.73)	0.009
VEGF–D (pg/mL)	−21.54 (−43.3; 0.22)	0.052
VEGFR3 (pg/mL)	−24.6 (−66.14; 16.94)	0.246

a Sex.

The presence of blood vessels was further assessed with ERG immunohistochemistry (Figure 1E) and confirmed in hematoxylin-eosin staining. The analysis revealed that men presented a greater amount of blood vessels than women [0.37 ± 0.36 for men vs. 0.24 ± 0.24 for women (blood vessels/mm^2^)] (Figures 1E,F). Small or medium-sized blood vessels were the most prevalent among the AVs. Men's AVs had higher densities for small and medium-sized blood vessels than women's [0.43 ± 0.24 for men vs. 0.33 ± 0.28 for women (small vessels/total vessels); 0.53 ± 0.31 for men vs. 0.41 ± 0.22 for women (medium vessels/total vessels)]. There were no differences in hypertrophied vessels (arterioles) density between the sexes [0.03 ± 0.07 for men vs. 0.02 ± 0.05 for women (hypertrophied vessels/total vessels)] (Figures 1E–G). Multivariate analyses adjusting for confounders confirmed a significant lower density of blood vessels/mm^2^ (OR = −0.17, *p* = 0.01) and medium-sized vessels/total vessels in women AVs than in men (OR = −0.14, *p* = 0.05) (Table 2). No significant differences were reported for small and hypertrophic-sized vessels.

### The expression of both pro-angiogenic and anti-angiogenic factors was increased in AVs from men

**Figure 2:**
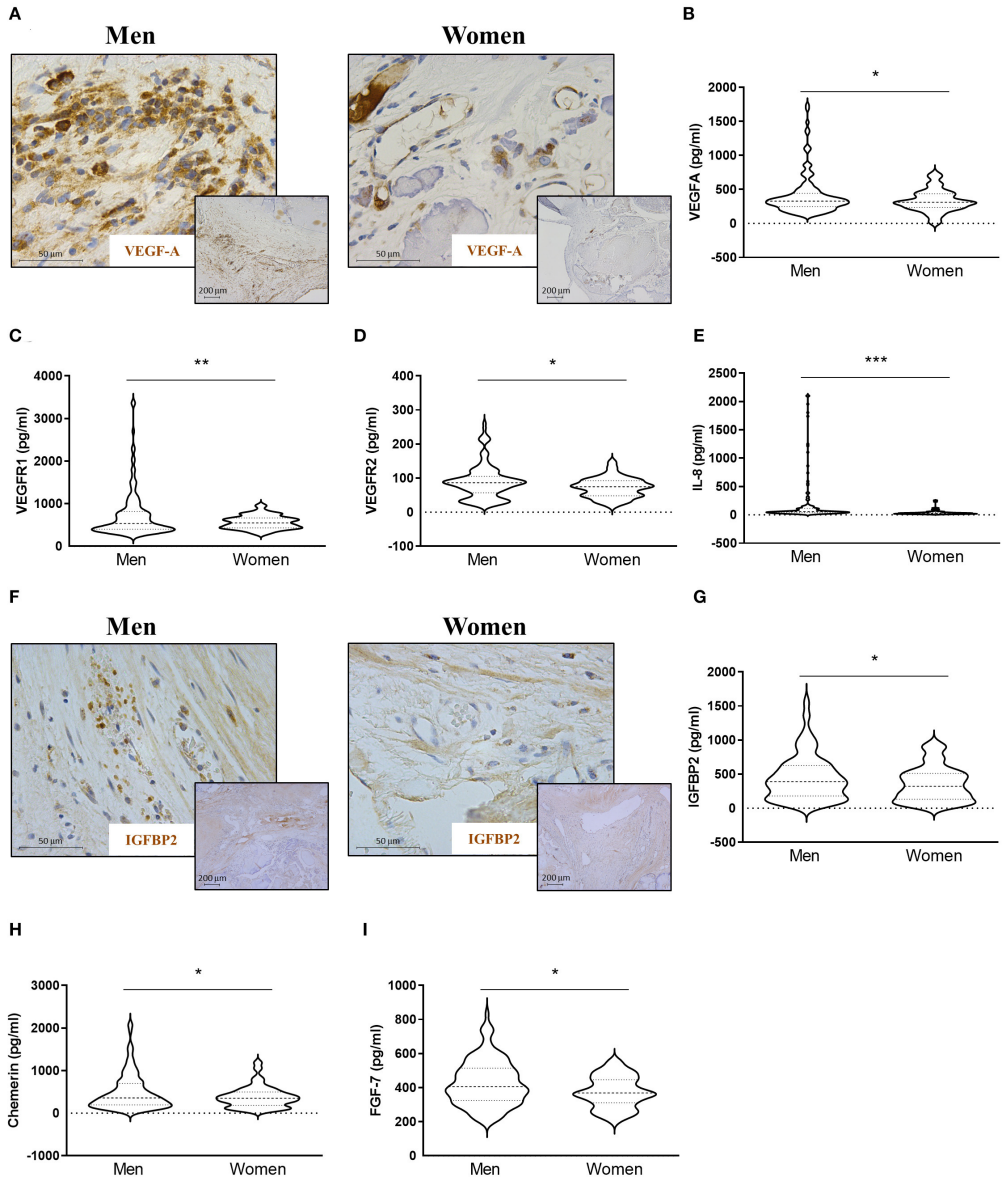
Sex differences in pro-angiogenic markers in AVs from AS patients. Representative microphotographs of AV sections from AS patients immunostained for VEGFA **(A)**. Protein expressions of VEGFA **(B)**, VEGFR1 **(C)**, VEGFR2 **(D)**, and IL-8 **(E)** in tissue homogenates from AVs of AS patients were measured by ELISA. Representative microphotographs are immunostained for IGFBP2 **(F)**. Protein expressions of IGFBP2 **(G)**, chemerin **(H)**, and FGF-7 **(I)** were measured by ELISA. VEGF: vascular endothelial growth factor; IL, interleukin; IGFBP, insulin-like growth factor binding protein. *N* = 132 for men and *N* = 86 for women by ELISA. *p < 0.05 vs. men, **p < 0.01 vs. men, ***p < 0.001 vs. men.

The expression of VEGF-A was increased in AVs from men as compared to women as evidenced by the representative immunostaining displayed in Figure 2A and its further quantification [413 ± 300 for men vs. 335 ± 164 for women (pg/ml)] (Figure 2B). In addition, VEGFR1 [744 ± 562 for men vs. 566 ± 157 for women (pg/ml)] (Figure 2C) and VEGFR2 [89 ± 50 for men vs. 72 ± 29 for women (pg/ml)] (Figure 2D) levels were higher in men-derived AVs than in women. Additional pro-angiogenic factors were assessed in AV from patients with AS. The expression of IL-8 was increased in men's AVs as compared to women's [194 ± 407 for men vs. 39 ± 39 for women (pg/ml)] (Figure 2E). Both immunostaining and protein quantification of IGFBP2 revealed a consistent increment in AVs from men [450 ± 331 for men vs. 359 ± 254 for women (pg/ml)] (Figures 2F,G). Chemerin expression was also significantly augmented in men's AVs [494 ± 422 for men vs. 382 ± 253 for women (pg/ml)] (Figure 2H). Levels of FGF-7, other known angiogenic growth factor, were similarly higher in AVs from men as compared to women [427 ± 132 for men vs. 373 ± 91 for women (pg/ml)] (Figure 2I). All the protein expression levels were measured by ELISA. Additional multivariate analyses corroborated that IL-8 and FGF7 expression was lower in women AVs than in men counterparts (OR = −179.48, *p* = 0.001; OR = −64.95, *p* = 0.001, respectively) after adjusting for confounders (age, statins treatment, and total cholesterol). Statistical trends were reported for VEGF-A, VEGFR1, VEGFR2, and IGFBP2 but they did not reach statistical significance (Table 2).

**Figure 3:**
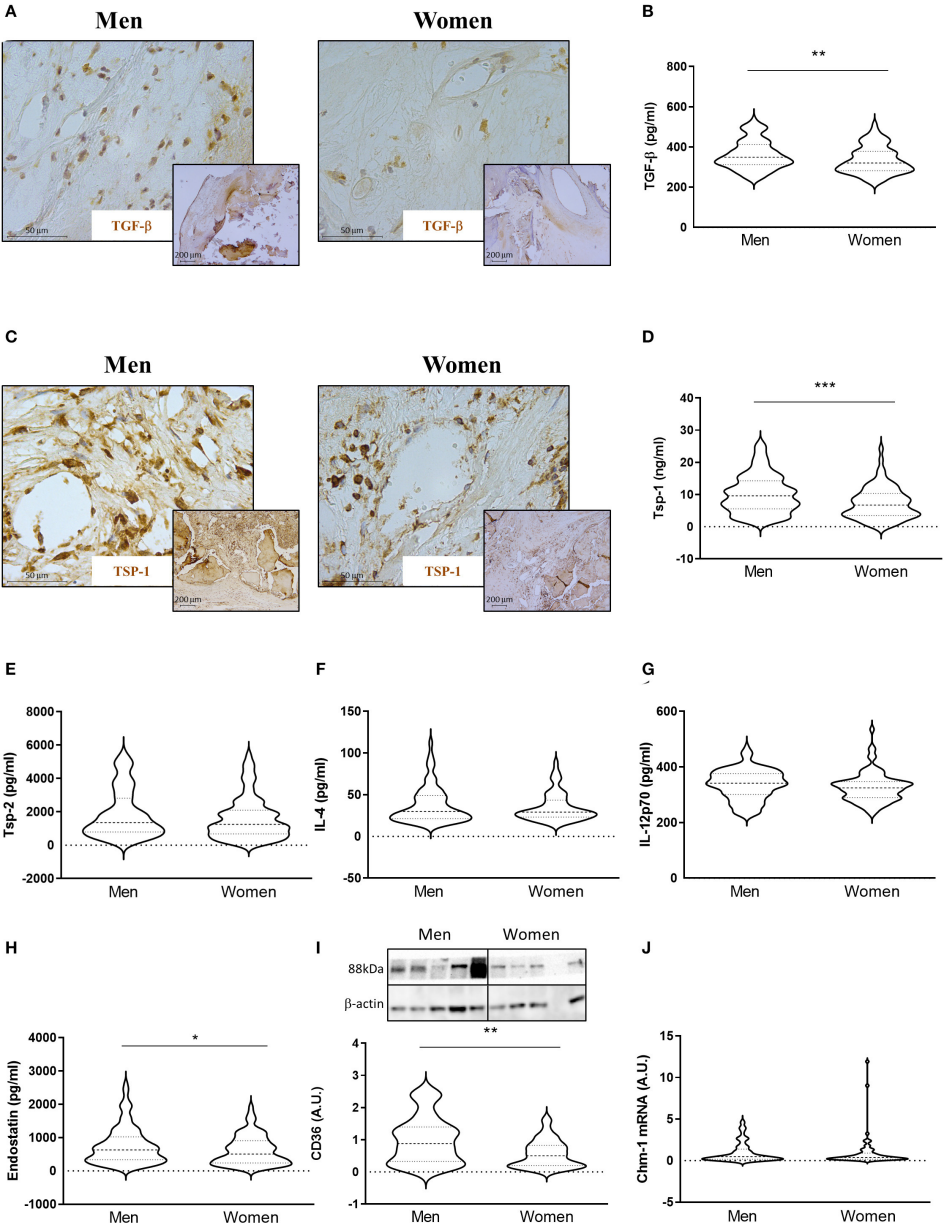
Sex differences in anti-angiogenic markers in AVs from AS patients. Representative microphotographs of AV sections from AS patients immunostained for TGF-β **(A)** and Tsp-1 **(C)**. Protein expressions of TGF-β **(B)**, Tsp-1, Tsp-2, IL-4, IL-12p70, and endostatin **(D–H)** in tissue homogenates from AVs of AS patients were measured by ELISA. Protein expression levels of CD36 by WB **(I)** and mRNA levels of Chm-1 **(J)** in AVs of AS patients. TGF-β, transforming growth factor-beta; Tsp, thrombospondin; IL, interleukin; Chm-1, chondromodulin-1. Different sample sizes were assayed depending on the analytical methods used. *N* = 132 for men and *N* = 86 for women by ELISA. *N* = 42 men and *N* = 43 women by WB. *N* = 11 men and *N* = 62 women by RT-qPCR. *p < 0.05 vs. men, **p < 0.01 vs. men, ***p < 0.001 vs. men.

Of special interest, TGF-β, a pleiotropic protein that can be either pro- or anti-angiogenic, (34) was also elevated in AVs from men [361 ± 72 for men vs. 335 ± 67 for women (pg/ml)] (Figures 3A,B) by immunostaining and ELISA. Such lower expression of TGF-β in women compared to men AVs was further confirmed by multivariate analyses after adjusting for the confounding factors stated above (OR = −57.14, *p* < 0.001).

We next investigated the expression of anti-angiogenic molecules. Immunohistochemical analyses showed higher expression of Tsp-1 in AVs from men (Figure 3C). This finding was further confirmed using quantitative analysis. Accordingly, AVs from men presented increased levels of Tsp-1 [10 ± 6 for men vs. 7 ± 5 for women (ng/ml)] (Figure 3D), whereas no changes were found in Tsp-2 expression [1897 ± 1446 for men vs. 1621 ± 1266 for women (pg/ml)] (Figure 3E). While IL-4 [38 ± 22 for men vs. 35 ± 17 for women (pg/ml)] (Figure 3F) and IL-12p70 [337 ± 54 for men vs. 327 ± 53 for women (pg/ml)] (Figure 3G) levels were similar in AVs from men and women, endostatin expression was elevated in men AVs as compared to women's [761 ± 557 for men vs. 616 ± 434 for women (pg/ml)] (Figure 3H). All of the previously mentioned markers were measured by ELISA. The expression of the scavenger receptor CD36 was also higher in men's AVs [1 ± 0.72 for men vs. 0.56 ± 0.42 for women (A.U.)] (Figure 3I) in WB. Similar mRNA levels of Chondromodulin-1 (Chm-1) were found in AVs in men and women [0.07 ± 0.08 for men vs. 0.07 ± 0.13 for women (A.U.)] (Figure 3J). Multivariate analyses adjusting for confounders also corroborated a significant downregulated expression of Tsp-1 (OR = −3.85, *p* < 0.001), IL-12p70 (OR = −19.24, *p* = 0.014), endostatin (OR = −197.81, *p* = 0.014), and CD36 (OR = −0.41, *p* = 0.014), with a statistical but not significant trend for Tsp-2 (OR = −368.12, *p* = 0.087), in women AVs as compared to men's (Table 2).

### AVs from men exhibited increased lymphangiogenesis

**Figure 4:**
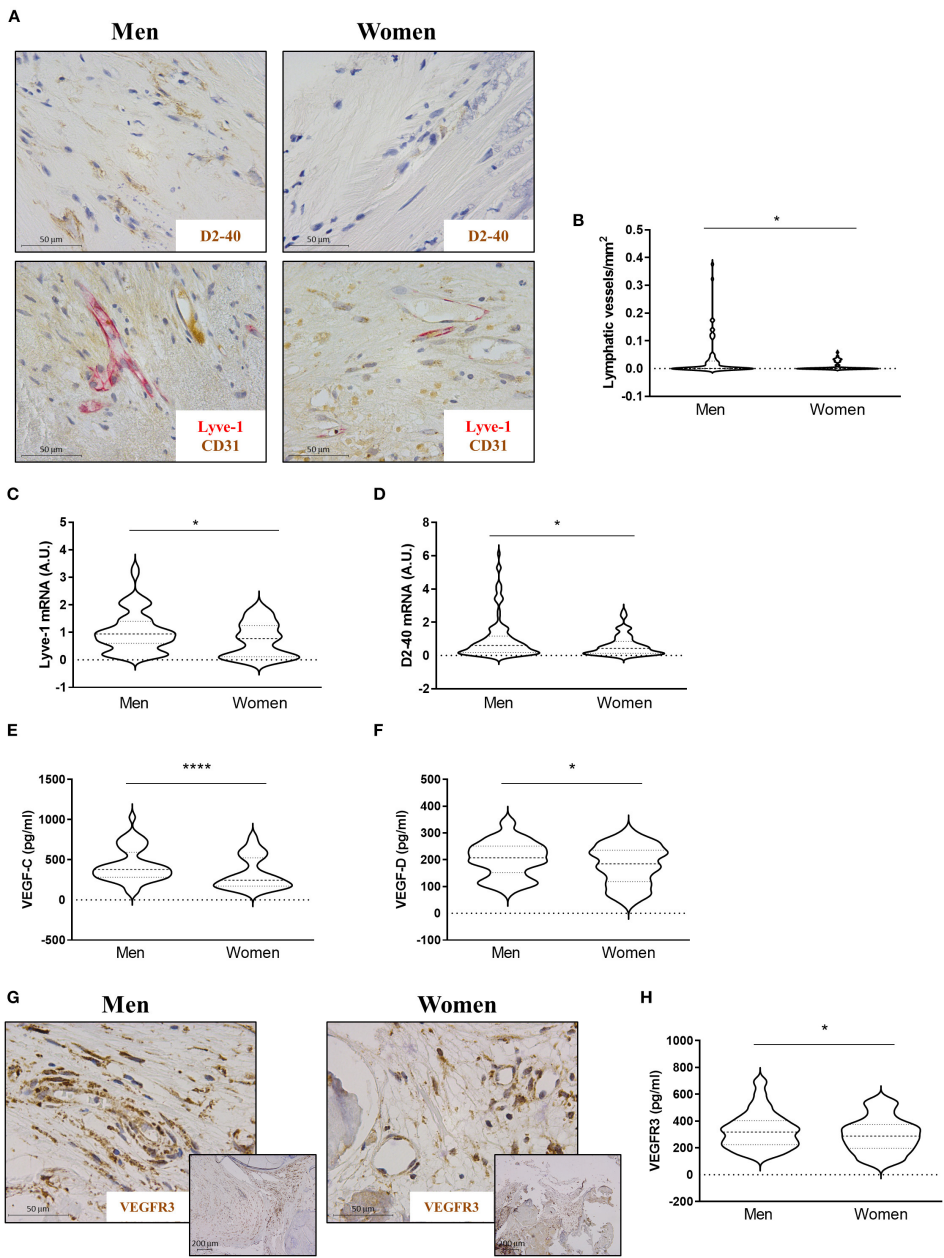
Sex differences in lymphangiogenesis in AVs from AS patients. Representative microphotographs of AV sections from AS patients immunostained for D2-40 and Lyve-1/CD31 **(A)**. Total lymphatic vessel density in AVs from men and women was measured by D2-40 and Lyve-1/CD31 immunostaining **(B)**. mRNA expression levels of Lyve-1 **(C)** and D2-40 **(D)** in AVs of AS patients. Protein expressions of VEGF-C and VEGF-D in tissue homogenates from AVs of AS patients were measured by ELISA **(E,F)**. Representative microphotographs are immunostained for VEGFR3 **(G)**. Protein expression of VEGFR3 **(H)** in whole AV tissue was measured by ELISA. VEGF: vascular endothelial growth factor. Different sample sizes were assayed depending on the analytical methods used. *N* = 99 for men and *N* = 69 for women by histology. *N* = 116 for men and *N* = 70 for women by RT-qPCR. *N* = 133 for men and *N* = 87 for women by ELISA. *p < 0.05 vs. men, ****p < 0.0001 vs. men.

The presence of lymphatic vessels in AVs was assessed with D2-40 and Lyve-1/CD31 (Figure 4A). Lymphatic vessels were found in 46.15% of the stenotic AVs, with no significant differences between men (48.33%) and women (43.18%). The number of lymphatic vessels per sample preparation was higher in AVs from men than in women [0.033 ± 0.07 for men vs. 0.008 ± 0.02 for women (lymphatic vessels/mm^2^)] (Figure 4B). In line with these results, the quantification of the mRNA of Lyve-1 [1.02 ± 0.73 for men vs. 0.73 ± 0.62 for women (A.U.)] and D2-40 [0.035 ± 0.043 for men vs. 0.021 ± 0.022 for women (A.U.)] showed an increase in men's AVs (Figures 4C,D). The expression levels of the lymphangiogenic molecules VEGF-C [434 ± 207 for men vs. 326 ± 198 for women (pg/ml)] and VEGF-D [203 ± 66 for men vs. 180 ± 72 for women (pg/ml)] were higher in AVs from men as compared to women by ELISA (Figures 4E,F). VEGFR3 immunostaining showed greater expression in neovessels in AVs from men as compared to women's AVs (Figure 4G). Accordingly, VEGFR3 ELISA expression was augmented in whole AV tissue from men with AS [333 ± 136 for men vs. 296 ± 130 for women (pg/ml)] (Figure 4H). Importantly, multivariate analyses adjusting for confounding factors further confirmed a significant lower density of lymphatic vessels (OR = −0.02, *p* = 0.045) in women AVs than in men (OR = −0.14, *p* = 0.05) (Table 2). Moreover, the lower expression in women AVs of the lymphangiogenic markers D2-40 and VEGF-C was validated by multivariate analyses after adjusting for confounding factors (OR = −0.01, *p* = 0.041; and OR = –79.05, *p* = 0.009, respectively), with a statistical trend for VEGF-D (OR = −21.54, *p* = 0.052) (Table 2).

### Production of pro-angiogenic, anti-angiogenic, and lymphangiogenic factors in human aortic VICs isolated from men and women with AS

**Figure 5:**
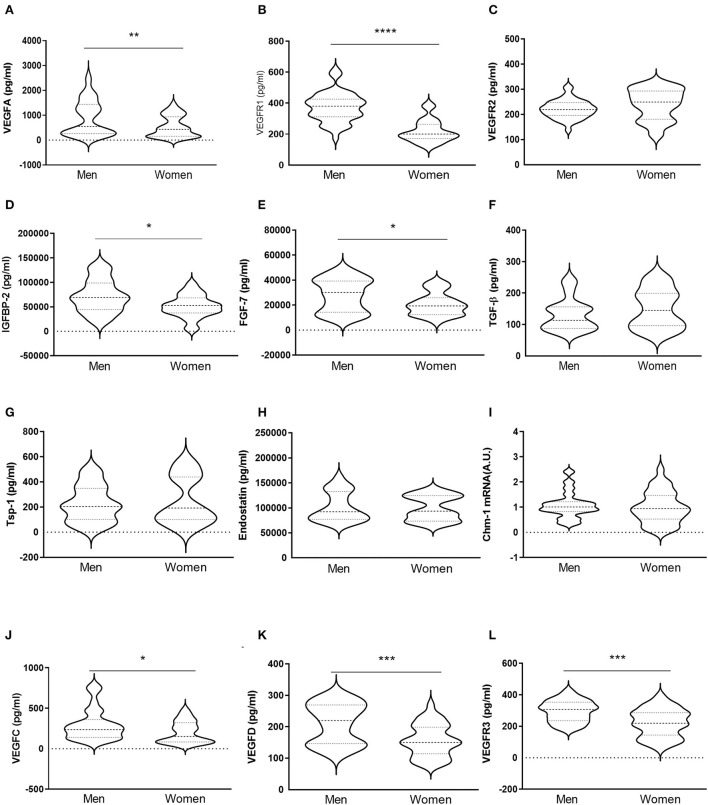
*In vitro* sex-comparative analysis of pro-angiogenic, anti-angiogenic, and lymphangiogenic markers in human aortic VICs. Protein expression of pro-angiogenic factors VEGF-A, VEGFR1, VEGFR2, IGFBP-2, and FGF-7 in VICs isolated from male and female-derived AVs from AS patients **(A–E)** measured from the supernatant by ELISA. Protein levels of TGF-β, Tsp-1, and endostatin in VICs from AVs of AS patients **(F–H)** were determined from the supernatant by ELISA. mRNA expression levels of Chm-1 **(I)**. Protein expression of lymphangiogenic factors VEGF-C, VEGF-D, and VEGFR3 **(J–L)** in the supernatant by ELISA. VEGF: vascular endothelial growth factor; IGFBP: insulin-like growth factor binding protein; FGF: fibroblast growth factor; TGF-β: transforming growth factor-beta; Tsp: thrombospondin; Chm-1: chondromodulin-1. *N* = 24 for men and *N* = 12 for women. *p < 0.05 vs. men, **p < 0.01 vs. men, ***p < 0.001 vs. men, ****p < 0.0001 vs. men.

VICs were isolated from male- or female-derived AVs from AS patients. Our results showed that VICs from men presented higher expression of VEGF-A [891 ± 706 for men vs. 541 ± 423 for women (pg/ml)] (Figure 5A) and VEGFR1 (375 ± 94 for men vs. 218 ± 72 for women [pg/ml)] (Figure 5B) as compared to VICs isolated from women. However, VEGFR2 levels did not differ between male and female VICs [221 ± 37 for men vs. 241 ± 61 for women (pg/ml)] (Figure 5C). An increase in IGFBP-2 secretion was found in men's VICs as compared to women's [71776 ± 33455 for men vs. 51004 ± 22946 for women (pg/ml)] (Figure 5D). Pro-angiogenic FGF-7 was higher in men's VICs supernatant as compared to women's [27926 ± 13059 for men vs. 20776 ± 9119 for women (pg/ml)] (Figure 5E). The expression of TGF-β [132 ± 51 for men vs. 151 ± 62 for women (pg/ml)], Tsp-1 [227 ± 145 for men vs. 248 ± 188 for women (pg/ml)], endostatin [101930 ± 29452 for men vs. 99215 ± 24532 for women (pg/ml)], and Chm-1 [1.08 ± 0.52 for men vs. 0.99 ± 0.63 for women (A.U.)] did not differ between men- and women-derived VICs (Figures 5F-I). Interestingly, VICs isolated from men exhibited higher levels of the lymphangiogenic factors VEGF-C [298 ± 193 for men vs. 202 ± 134 for women (pg/ml)], VEGF-D [210 ± 64 for men vs. 156 ± 51 for women (pg/ml)], and VEGFR3 [296 ± 68 for men vs. 222 ± 89 for women (pg/ml)] (Figures 5J-L). All these markers were measured by ELISA on cell supernatants.

## Discussion

Our data demonstrate for the first time the sex-related differences in angiogenesis and lymphangiogenesis in AVs and in isolated VICs from men and women with AS. Results presented here show that AVs from men exhibit higher densities of blood and lymphatic neovessels than those from women, even after adjusting for confounding factors, such as age, total cholesterol, and statin intake. In men's AVs and VICs, there is an angiogenic switch characterized by an imbalance between pro- and anti-angiogenic factors, with subsequent activation of angiogenesis. Moreover, our *in vitro* results using primary VICs isolated from AS patients reinforce the idea that VICs are a major player regulating neovascularization of the AV in AS with a marked sex-specific pattern. Thus, male-derived VICs exhibited higher pro-angiogenic and lymphangiogenic capacity than female-derived VICs.

The information about the relevance of angiogenesis in AVs is scarce, even more for lymphangiogenesis. Previous studies have shown that both phenomena are induced in AVs from AS patients (15, 17, 18), likely contributing to the harmful accumulation of inflammatory cells and calcification. The loss of avascularity in the AV seems to respond to the enhanced requirement of oxygen supply within the extensive thickening and calcification occurring during the progression of AS. The extent of neovascularization correlates with the burden of AS (8, 9) and is associated with osteogenesis and calcification in the cardiovascular territory (8, 10). Small and medium-sized vessels and arterioles have been previously described in AVs from AS patients (18). Our results also reveal that small and medium-sized neovessels are the predominant forms of neovascularization in the AV of patients with AS, especially in men compared to women. The presence of both blood and lymphatic vessels was associated with lymphocytic infiltrates in 86% of cases in a small cohort of AS patients (17). In rheumatic valves, the presence of inflammatory infiltrates as well as the expression VEGF have been described in areas of neoangiogenesis (9). The relationship between angiogenesis and inflammation remains, however, controversial. On one hand, it has been proposed that inflammation could precede and promote angiogenesis (35). On the other hand, it has been described that elastic fiber fragmentation leads to aberrant angiogenesis and which precedes inflammation in aortic valve diseases (15). In our study, neovessels were also located near the inflammatory foci, the calcification nodules, and the endothelial layer. Both processes have been demonstrated to be over-represented in AVs among men than women in a similar cohort (6). The network of neovessels may facilitate rapid transit of inflammatory cells and pro-calcifying molecules. However, all the patients present similar severity of AS, being difficult to conclude whether angiogenesis could precede or follow inflammatory infiltration.

The source of neovessels has been a matter of debate in the last years. Initially, it was postulated that one source of this neovascularization could come from valve endothelial cells (VECs) (17). Both VICs and VECs in coculture could undergo pericyte differentiation and angiogenic sprouting, respectively (19). Recently, it has been confirmed that VICs isolated from AS patients are angiogenic cells and could differentiate into perivascular cells and secrete VEGF-A (20). Herein, we extend those findings by demonstrating that VEGF-A and other factors that contribute to angiogenesis are found in conditioned media from VICs isolated from diseased patients, with marked sex differences. Up to now, only two studies described sex differences in angiogenesis potentially relevant to the progression of AS but based on isolated VICs from porcine AVs (26, 27). McCoy and co-workers were the first to describe that VEGFR2 levels were five-fold higher in isolated VICs from male as compared to female cells (26). These results are consistent with our observations describing that male AVs expressed higher levels of VEGFR2 relative to female counterparts, although these findings have not been seen *in vitro*. Nevertheless, male VICs exhibited higher VEGF-A, VEGFR1, IGFBP-2, and FGF-7 than female VICs, without major changes in the expression of anti-angiogenic molecules. Recently, Nelson and coworkers evidenced that male quiescent VICs secrete lower levels of VEGF-A than female VICs, while VEGF-A is upregulated upon VIC activation and reaches expression levels comparable to female VICs (27). Interestingly, in our study, we evidenced that VEGF-A levels were higher in male VICs than in female's isolated from AS patients. The overall secretome and proteome of VICs isolated from end-stage stenotic AVs might show differences compared to those found in control non-diseased swine AV such as these used in previous publications (27).

Interestingly, TGF-β was overexpressed in male AVs, as it has been previously described by McCoy et al. (26). It is well known that TGF-β induces calcification and differentiation of VICs into myofibroblastic cells (36), so increased levels of this cytokine may be related to a greater degree of calcification in male patients (37) rather than to its antiangiogenic role. Concerning antiangiogenic factors, AVs from women exhibited reduced levels of Tsp-1 and endostatin as compared to men, with no differences in Tsp-2 expression. Tsps are highly conserved calcium-binding matricellular proteins regulating angiogenesis but also inflammation and extracellular matrix remodeling, and their contribution to such mechanisms might be different according to the sex. For instance, Tsp-1 is significant for initial fibro-inflammatory response and Tsp-2 for proliferative and remodeling phases within neoangiogenesis (38). Although the evidence suggesting an association between Tsp-1 and AS is limited, it seems that the expression of Tsp-1 is similar in fibrosclerotic and stenotic AVs compared to controls (39). Of note, Tsp-1 can activate TGF-β and bind CD36 to actively participate in fibrotic processes (40). Accordingly, women AVs exhibited lower TGF-β and CD36 and presented exacerbated extracellular matrix remodeling and low inflammatory profiles (6). In contrast, Tsp-2 levels were higher in fibrotic and stenotic AVs than in control ones (39). Tsp-2 has been associated with myofibroblast proliferation and neovascularization in the AV (39), both of which are also higher in men-derived AVs. However, our results did not show differences between the sexes. In contrast with previous publications, our study compares the expression profiles of Tsps in AVs from end-stage severe forms of AS rather than fibrosclerotic vs. stenotic AVs. That might explain, at least in part, the discrepancies among our results and previous publications. Other anti-angiogenic factor, endostatin, has been found to be increased in AS patients and associated to calcification (11). In consequence, its expression is decreased in women AVs, which presented lower degree of calcification for the same AS severity (6). It has been postulated that the presence of endostatin could indicate that anti-angiogenic processes are also activated during the development of AS (11) and in line with our results in men-derived AVs might fail to resolve the aberrant loss of avascularity during the progression of AS.

Lymphangiogenesis mainly consists of the growth of lymphatic vessels from pre-existing neovessels in which the endothelial cell component exposed to VEGF-C and -D may differentiate into lymphatic endothelial cells (41). Lymphatic vessel density was higher in men's AVs than in women's. Moreover, male AVs presented higher levels of Lyve-1, VEGF-C, VEGF-D, and VEGFR3 suggesting that this process could be decreased in women. Although VEGF-C levels were not found to be overexpressed in AS valves, there is evidence that it is secreted by VICs and that increased levels of it correlate with a greater transvalvular pressure gradient (18). Consistently, VICs from men exhibited higher secretion of VEGF-C, VEGF-D, and VEGFR3. Interestingly, increased amounts of VEGFR3 in men's AVs could explain the observed differences in the presence and localization of lymphatic vessels as it has been reported for control and stenotic AVs (18). A high expression of VIC-derived VEGF-C may recruit blood endothelial cells from the valvular angiogenic sprouts to differentiate into lymphatic endothelial cells (42).

The overall higher angiogenic and lymphangiogenic profiles in men compared to women may also contribute to the development of fibro-calcific phenotypes in AS in men. Endochondral bone formation is regulated by systemically and locally acting growth factors, such as VEGFs and their receptors. VEGF-A has been proven to regulate bone formation toward the activation of angiogenesis (43). We and others have found increased inflammation, osteogenesis, and calcification in men compared to women (6, 26, 44). VIC-derived pro-angiogenic and pro-lymphangiogenic morphogens may sustain early AV thickening and sclerosis. In men-derived VICs, with higher osteoblastic profiles, these molecules may further promote osteoblast differentiation and AV-to-bone replacement.

In conclusion, angiogenesis and lymphangiogenesis are different in men and women with AS. AVs from men presented more neovessels as well as an imbalance between pro- and anti-angiogenic factors. Our study provides new molecular and cellular insights on the pathogenesis of AS with relevant sex-specific signatures that might be clinically relevant to the development of sex-tailored therapeutic strategies.

## Limitations

This study had several limitations. First, we have not been able to perform the histological staining and characterization of vessels in the total sample, which could have provided us with greater robustness in our Histopathological results with respect to the molecular analyses. Second, we only have paraffin-embedded tissue, which facilitates the sectioning of the calcified tissue and better morphology, but we lose information due to the inability to label certain molecules. Third, we have not explored the role of VECs in angiogenesis and lymphangiogenesis. Future *in vitro* studies comparing the activation of angiogenic and lymphangiogenic pathways in VICs undergoing osteogenic differentiation would be appropriate to parallel findings in end-stage clinical samples. Moreover, it would be useful to search for therapeutic targets capable to revert or prevent these pathological events.

## Data availability statement

The original contributions presented in the study are included in the article/Supplementary material, further inquiries can be directed to the corresponding authors.

## Ethics statement

The study protocol involving human participants was approved by the Comité Ético de Experimentación Clínica, Gobierno de Navarra - Departamento de Salud (Ethics numbers 17/2013 and PI2019/59) and complied with ethical standards of the Declaration of Helsinki. Written informed consent to participate in this study was obtained from all participants.

## Author contributions

NL-A and EJ conceived and designed the study. LM, EM-N, MG, JV, AN, VA, AG-P, AF-C, AG, VA, and RS performed the data. LM, EM-N, MG, JV, AN, VA, AG-P, AF-C, AG, VA, RS, EJ, and NL-A analyzed and interpreted the data. LM, EJ, and NL-A led the design and drafted the article. All authors contributed to the article and approved the submitted version.

## Funding

This work was supported by a Miguel Servet contract (CP13/00221) and by Fondo de Investigaciones Sanitarias (PI18/01875; PI21/00280) from the Instituto de Salud Carlos III - FEDER. LM was supported by a PFIS Ph.D. studentship (FI19/00302). EJ was supported by a Sara Borrell postdoctoral fellowship (CD19/00251). EM-N was supported by a Margarita Salas postdoctoral fellowship (ULL-MS-P14). MG was supported by a Miguel Servet Foundation Ph.D. studentship.

## Conflict of interest

The authors declare that the research was conducted in the absence of any commercial or financial relationships that could be construed as a potential conflict of interest.

## Publisher's note

All claims expressed in this article are solely those of the authors and do not necessarily represent those of their affiliated organizations, or those of the publisher, the editors and the reviewers. Any product that may be evaluated in this article, or claim that may be made by its manufacturer, is not guaranteed or endorsed by the publisher.

## Abbreviations

AS: aortic stenosis
AU: arbitrary units
AV: aortic valve
BMP: bone morphogenetic protein
CCL2: C-C motif chemokine ligand 2
Chm-1: chondromodulin-1
ERG: erythroblast transformation-specific related gene
FBS: fetal bovine serum
FGF: fibroblast growth factor
IL: interleukin
IGFBP-2: insulin-like growth factor-binding protein-2
TGF-β: tissue growth factor-beta
Tsp: thrombospondin
VEGF: vascular endothelial growth factor
VEC: valve endothelial cell
VIC: valve interstitial cell
WB: western blot.
